# Supplementary figures and images for: Pregnant women’s perceptions of the COVID-19 vaccine: A French survey
Source: PLoS One. 2022 Feb 7;17(2):e0263512. doi: 10.1371/journal.pone.0263512 (PMC8820613; doi:10.1371/journal.pone.0263512)

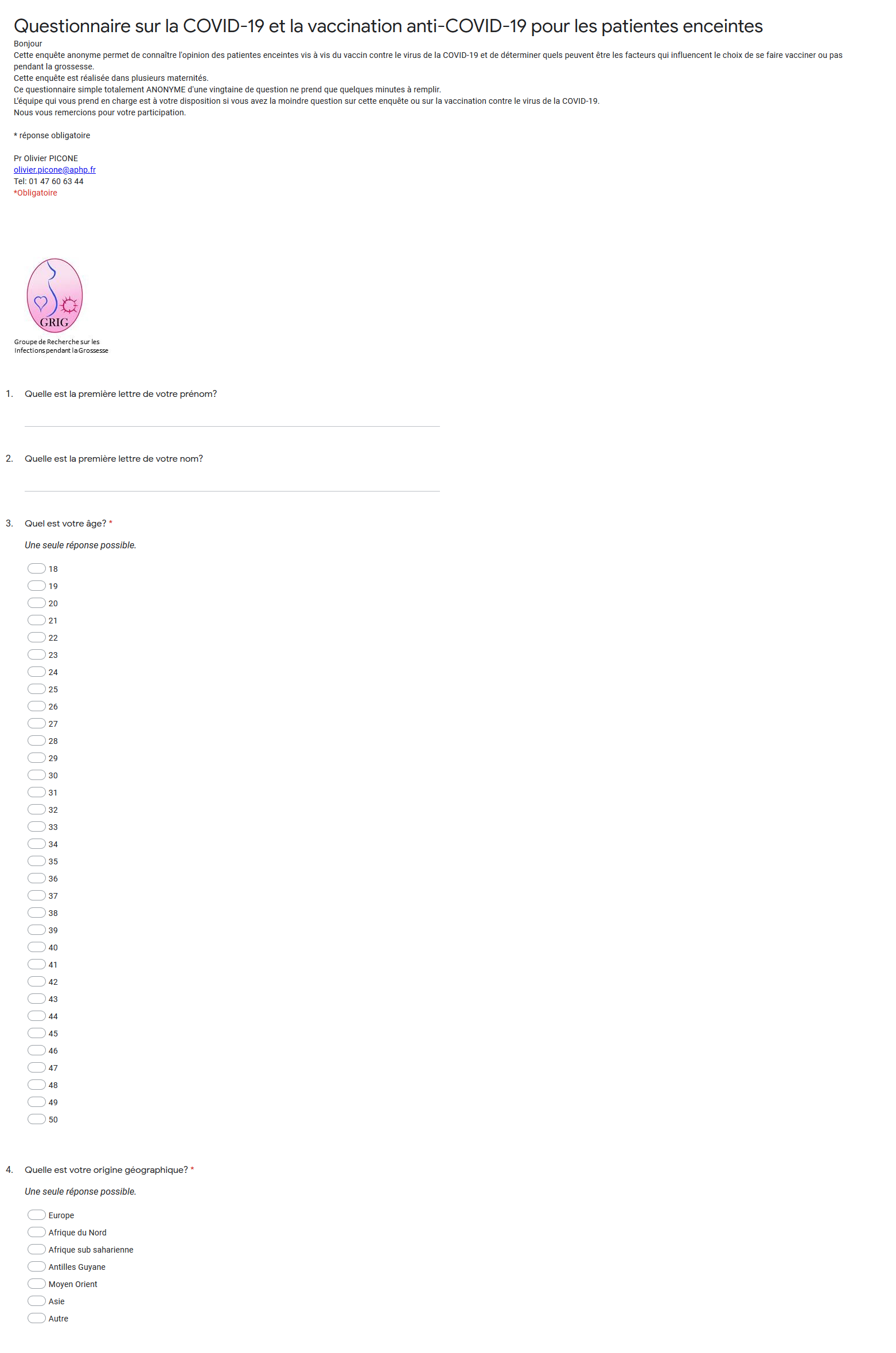

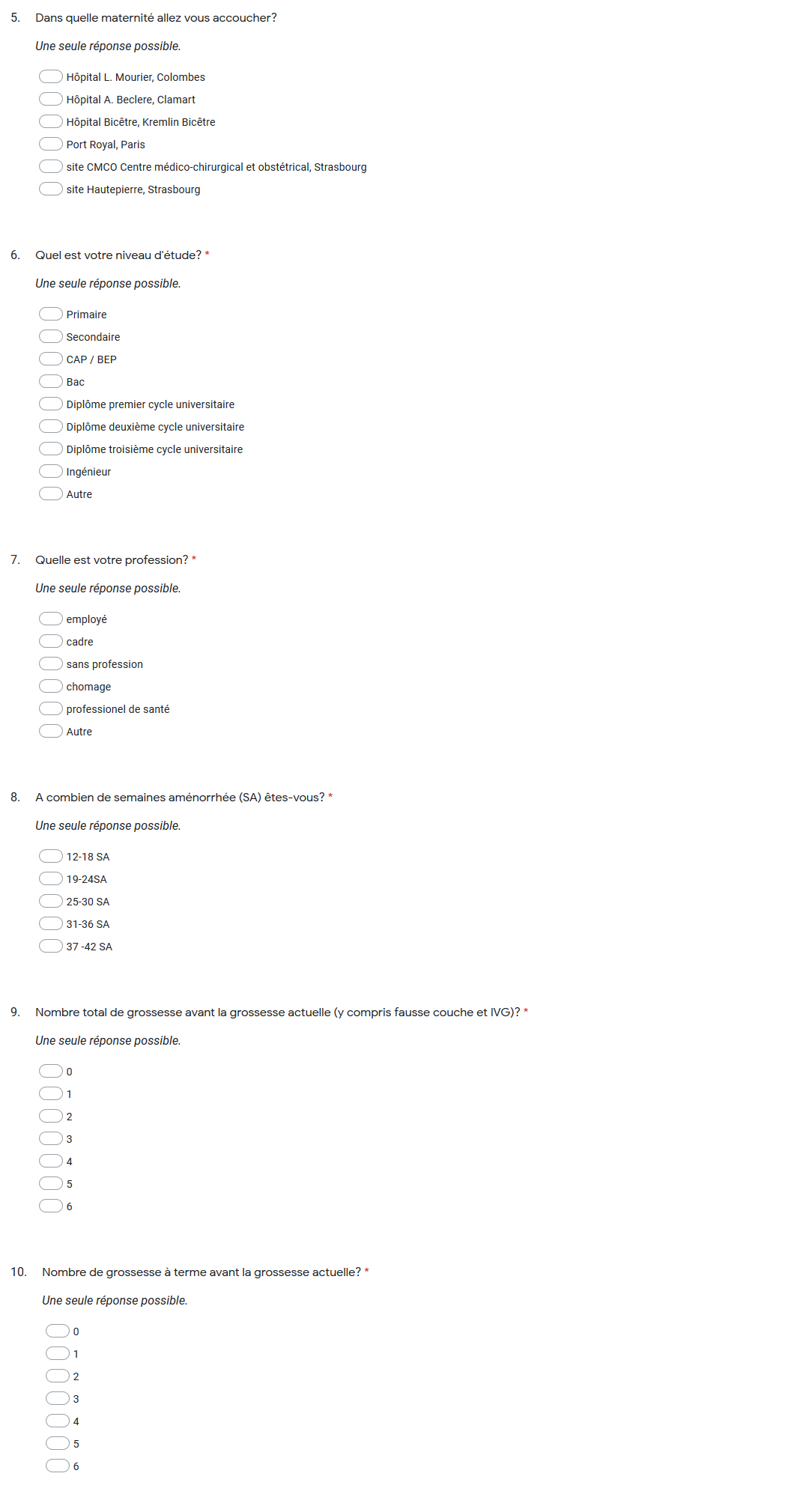


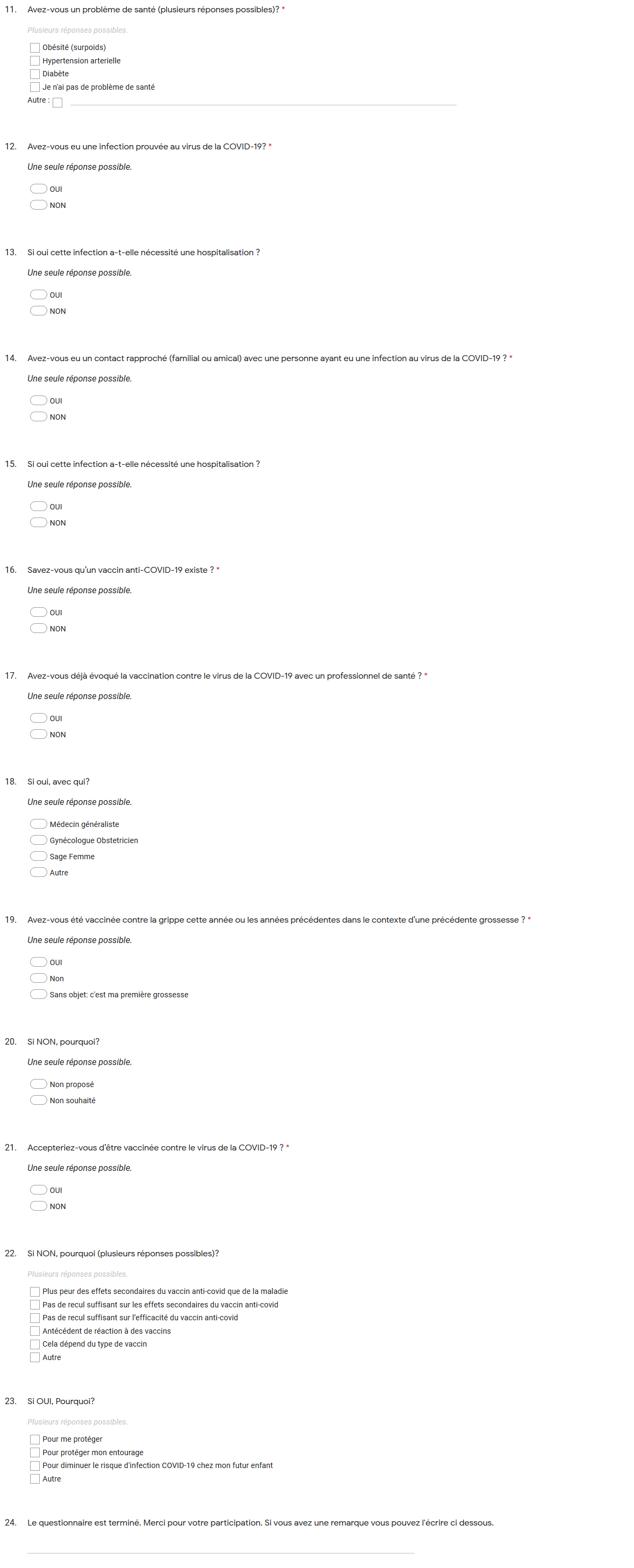

Supplement: S1 File — (DOCX) [file pone.0263512.s001.docx]
