# Supplementary material for: Pregnant women’s perceptions of the COVID-19 vaccine: A French survey
Source: PLoS One. 2022 Feb 7;17(2):e0263512. doi: 10.1371/journal.pone.0263512 (PMC8820613; doi:10.1371/journal.pone.0263512)
Supplement: Attachment — Submitted filename: Reviewer 1_20211116_CCL.docx [file pone.0263512.s002.docx]

| **Comments** | **Response** |
| --- | --- |
| **Reviewer 1** |  |
| Table 1 should be break down into 4 separate tables Like table 1: Pregnant women's sociodemographic characteristics and perceptions of the SARS-CoV-2 vaccine table 2: Pregnancy characteristics and perceptions of the SARS-CoV-2 vaccine table 3: Exposure to COVID-19 and perceptions of the SARS-CoV-2 vaccine table 4: Exposure and knowledge of vaccination and perceptions of the SARS-CoV-2 vaccine | Thank you for your comment.  Indeed, separating the table will allow a better understanding of the data. We have therefore modified the text. |
| Relationship between previous exposure to COVID-19 and Vaccine acceptance should be highlighted both in results and discussion section | Thank you for your comment.  We have emphasized this part in the result and discussion.  Result: “In contrast, had a proven SARS-CoV-2 infection was not associated with acceptance rates (p=0.570).”  Discussion: “Our results do not show a significant difference between history of exposure to SARS-Cov-2 and acceptance rate to be vaccinated. This may be related to the fact that patients with a previous SARS-CoV-2 infection believe they are still protected. Thus, patients with severe disease requiring hospitalization appear to be more likely to be vaccinated (5,9% vs 2,1%), although due to the small number of hospitalized patients, statistical analysis could not be performed.” |
| **Reviewer 2** |  |
| Firstly, I feel that for readers to appreciate this study it is important to add further details about the context in which the study was carried out. As you are well aware the period in which the study took place was to say the least fairly turbulent with regard to how vaccination was being reported in the French and European media (not to mention social media) and indeed also on a more specialist front with respect to advice being offered by different agencies and expert bodies. AS you rightly point out this differed in emphasis concerning vaccination for pregnant women. It is well-accepted that cross-sectional studies are undertaken at a point of time or over a short period of time. Nevertheless although single individuals are being questioned with regard to their attitudes at a single point of time, the context in which individual attitudes are being investigated was in this particular case rather labile. Notably with regard to Astra zenica. Even if this was not the vaccine of choice against Covid one cannot help but feel that it is difficult not to take into account in your discussion of results and possible limitations  Astra zenica was at the centre of a media and Health agency storm in early March. After having been launched on 25Th February 2021, following reports of rare adverse effects, it was suspended for use on 15/03 2021 and then reauthorized on 19th March. Could survey responses be influenced by such a labile context? Further more advice being offered as the authors duly point out varied between agencies and scientific bodies at the time. Thus advice from different agencies differed at least in strength of recommendation for pregnant women to get vaccinated. This also may have fueled the prevailing uncertainty. and although perhaps not as media worthy, was as you are well-aware aired in the medical press accessible on line. It is easy to imagine that this turbulent context could have had an influence on attitudes, even if subsequently Astra Zenica was not recommended for administration to pregnant women. The vaccines of choice being Moderna and BioNtech Pfizer. Evoking context raises a big issue in social psychology . ( See Schwarz,2007) Should attitudes be considered as being dispositional i.e. people have attitudes and they remain rather stable. An observers perspective. This downplays context. Or rather as construals, Evaluative judgements constructed in situations by taking into account the context and possible circulating information An actor’s perspective This highlights how attitudes can be context sensitive. On could also suggest that a changing epidemiological context may affect how people feel about Covid reducing or augmenting perceived risk. Could attitudes be affected or not affected, by such events and differences in advice? How could this affect the cross-sectional study even if the period over which the study was conducted was relatively short? At the time vaccination was not yet available to all of the pregnant women Furthermore as far as I can ascertain attitudes to different vaccines understandably were not taken into consideration within the questionnaire. These matters I feel merit discussion in the paper or at least cited as a possible limitation. | Thank you for your comment. Indeed, the media context in which this study was conducted probably influenced the results. We have therefore discussed this point in the limitations.  Discussion: “ Our study was conducted between February 18 to April 5 2021, during a turbulent period regarding the media coverage of vaccination in the French and European country. In particular, the Astra Zeneca vaccine, although never recommended for pregnant women, has been the source of much concern following reports of rare adverse events. Thus, survey responses could be influenced by such this labile context.” |
| A second difficult issue concerns clinical advice and its relationship to research evidence. This deserves reflection. It makes good sense to offer advice to any person seeking vaccination, all the more so in the case of pregnant women. Furthermore as the authors point out probably the best people to give health information are the health professionals caring for the pregnant women themselves since trust in a clinical relationship is paramount. Nevertheless when advice by leading bodies and experts (including members of the research team who took an active role in the debate ) is differing how can such uncertainty be tackled in clinical practice? At what point does research evidence cease to be part of ‘science in the making’ and become ‘ready made science’ ( cf Science in Action, Latour) | Thanks you for your comment.  It is true that, especially in these unstable periods, where research advances very quickly, it is difficult to position oneself formally as a practitioner. Thus, it is the duty of learned societies to issue recommendations in order to clarify the management and advice to patients.  We have therefore discussed this point in the discussion  Discussion: “During this pandemic, the opinions of different organizations and experts could sometimes differ and be contradictory, making it difficult for practitioners to advise patients. Thus, it is the duty of the main learned societies to issue recommendations, making it possible to guide health professionals in order to have a univocal and clear discourse for patients.” |
| **Editor** |  |
| . Please ensure that your manuscript meets PLOS ONE's style requirements, including those for file naming. The PLOS ONE style templates can be found at  [https://journals.plos.org/plosone/s/file?id=wjVg/PLOSOne_formatting_sample_main_body.pdf](https://journals.plos.org/plosone/s/file?id=wjVg/PLOSOne_formatting_sample_main_body.pdf) and  [https://journals.plos.org/plosone/s/file?id=ba62/PLOSOne_formatting_sample_title_authors_affiliations.pdf](https://journals.plos.org/plosone/s/file?id=ba62/PLOSOne_formatting_sample_title_authors_affiliations.pdf) | We ensured that our manuscript meets PLOS ONE’s style requirements. |
| 2. Please review your reference list to ensure that it is complete and correct. If you have cited papers that have been retracted, please include the rationale for doing so in the manuscript text, or remove these references and replace them with relevant current references. Any changes to the reference list should be mentioned in the rebuttal letter that accompanies your revised manuscript. If you need to cite a retracted article, indicate the article’s retracted status in the References list and also include a citation and full reference for the retraction notice. | We ensured that our reference list is complete and correct. |
| 3. Please provide additional details regarding participant consent. In the ethics statement in the Methods and online submission information, please ensure that you have specified (1) whether consent was informed and (2) what type you obtained (for instance, written or verbal, and if verbal, how it was documented and witnessed). If your study included minors, state whether you obtained consent from parents or guardians. If the need for consent was waived by the ethics committee, please include this information. If you are reporting a retrospective study of medical records or archived samples, please ensure that you have discussed whether all data were fully anonymized before you accessed them and/or whether the IRB or ethics committee waived the requirement for informed consent. If patients provided informed written consent to have data from their medical records used in research, please include this information | We specified how the data collection agreement was obtained i.e. in writing when completing the questionnaire. No minors were included. |
| 4. Please include additional information regarding the survey or questionnaire used in the study and ensure that you have provided sufficient details that others could replicate the analyses. For instance, if you developed a questionnaire as part of this study and it is not under a copyright more restrictive than CC-BY, please include a copy, in both the original language and English, as Supporting Information. | Thank you for this comment.  We have added the original questionnaire as a supplement S1 Table |
| 5. Thank you for stating the following financial disclosure:  "the funders had no role in study design, data collection and analysis, decision to publish, or preparation of the manuscript."  At this time, please address the following queries:  a) Please clarify the sources of funding (financial or material support) for your study. List the grants or organizations that supported your study, including funding received from your institution.  b) State what role the funders took in the study. If the funders had no role in your study, please state: “The funders had no role in study design, data collection and analysis, decision to publish, or preparation of the manuscript.” c) If any authors received a salary from any of your funders, please state which authors and which funders. d) If you did not receive any funding for this study, please state: “The authors received no specific funding for this work.”  Please include your amended statements within your cover letter; we will change the online submission form on your behalf. | We have declared the financial disclosure |
| 6. In your Data Availability statement, you have not specified where the minimal data set underlying the results described in your manuscript can be found. PLOS defines a study's minimal data set as the underlying data used to reach the conclusions drawn in the manuscript and any additional data required to replicate the reported study findings in their entirety. All PLOS journals require that the minimal data set be made fully available. For more information about our data policy, please see [http://journals.plos.org/plosone/s/data-availability](http://journals.plos.org/plosone/s/data-availability). | We specified were the mininal data set underlying the results described in our manuscript can found. |
| 7. Please include a copy of Table S1 which you refer to in your text on page 4. | Of course, as mentioned in the response to comment 4, we have added the original questionnaire as a supplement. |
